# Supplementary material for: E3 Ubiquitin Ligase FBXO3 Drives Neuroinflammation to Aggravate Cerebral Ischemia/Reperfusion Injury
Source: Int J Mol Sci. 2022 Nov 7;23(21):13648. doi: 10.3390/ijms232113648 (PMC9658360; doi:10.3390/ijms232113648)
Supplement: Supplementary file 1 [file ijms-23-13648-s001.zip › ijms-1987289-supplementary.pdf]

Table S1. Relative primer sequences for siRNA of FBXO3 (rat)

Gene name	Primer sequences
si-231 (rat)	Forward: GGAAGAAUCAGUGCUGGAATT<br>Reverse: UUCCAGCACUGAUUCUUCCTT
si-857 (rat)	Forward: GCAACAACUGGAGAUAUUATT<br>Reverse: UAAUAUCUCCAGUUGUUGCTT
si-1211 (rat)	Forward: CCACGAUUCCAUAUGGCAUTT<br>Reverse: AUGCCAUAUGGAAUCGUGGTT
NC (rat)	Forward: UUCUCCGAACGUGUCACGUTT<br>Reverse: ACGUGACACGUUCGGAGAATT

Table S2. Relative primer sequences for siRNA of FBXO3 (mouse)

Gene name	Primer sequences
si-174 (mouse)	Forward: GCUAUGUUAGUCGAAGACUUATT<br>Reverse: UAAGUCUUCGACUAACAUAGCTT
si-480 (mouse)	Forward: CGGAUGAUUAUCGCUGUUCAUTT<br>Reverse: AUGAACAGCGAUAAUCAUCCGTT
si-1100 (mouse)	Forward: CCUGGAGUAGUCGGUGAAUUUTT<br>Reverse: AAAUUCACCGACUACUCCAGGTT
NC (mouse)	Forward: UUCUCCGAACGUGUCACGUTT<br>Reverse: ACGUGACACGUUCGGAGAATT

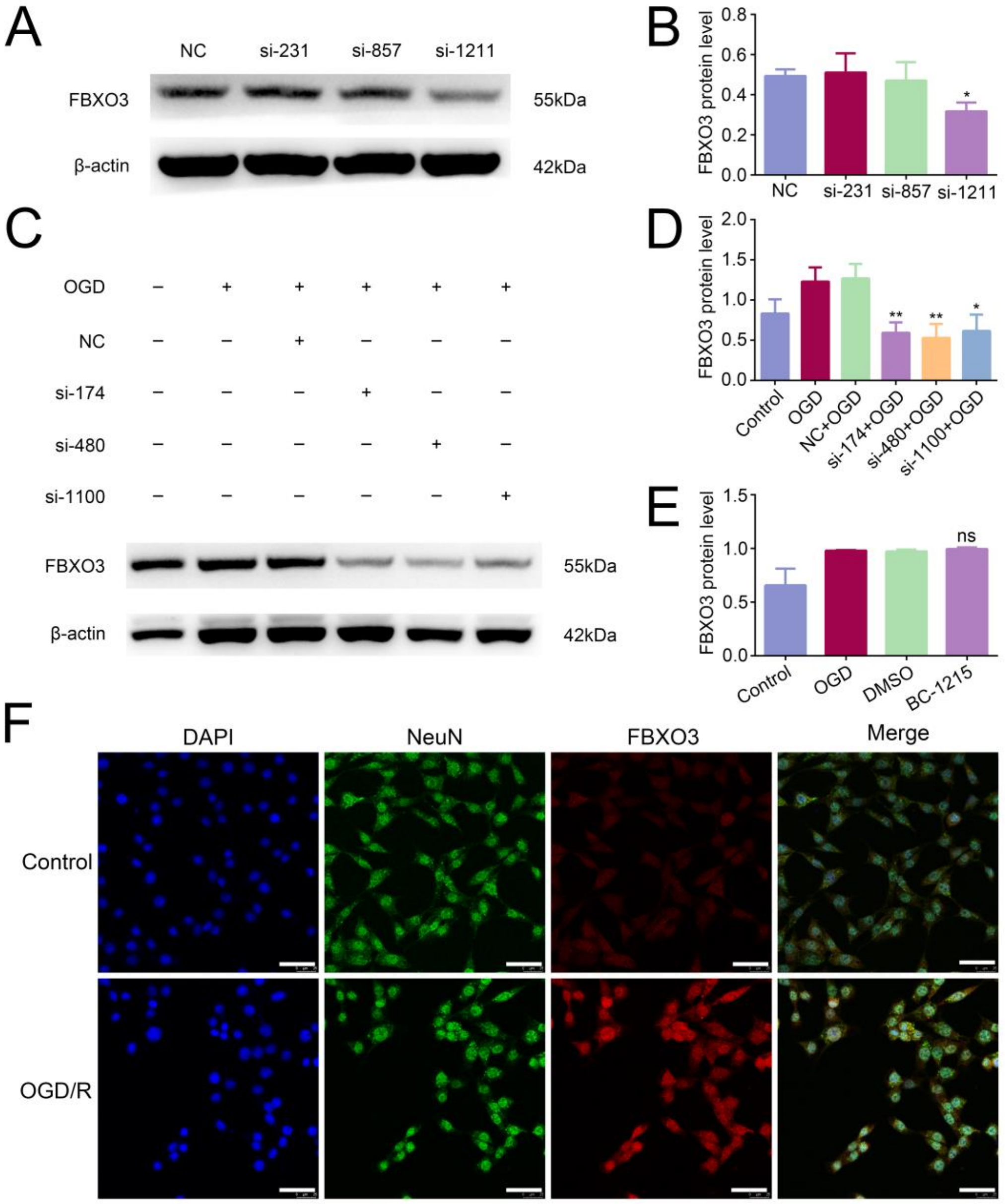

Figure S1. (A-B). Western blotting analysis of FBXO3 in the cortex of SD rats with different siRNA treatment. Si-1211 was chosen for relative experiments *in vivo* for its successful interference of FBXO3 protein expression. (C-D). Western blotting analysis of FBXO3 in HT22 cells at OGD 4 h/R 24 h with or without siRNA treatment. Si-480 was chosen for relative experiments *in vitro* for its successful interference of FBXO3 protein expression. (E). Analysis of FBXO3 protein level at OGD 4 h/R 24 h with DMSO or BC-1215 treatment. (F). IF colocalization of FBXO3 (red) along with NeuN (green) in HT22 cells with or without OGD/R treatment. Statistics for each group are expressed as mean ± SD ($n \geq 6$). *$p<0.05$, **$p<0.01$, vs. NC group or NC+OGD group.
